# The modern human aryl hydrocarbon receptor is more active when ancestralized by genome editing

**DOI:** 10.1073/pnas.2402159121

**Published:** 2024-05-13

**Authors:** Nelly Helmbrecht, Martin Lackner, Tomislav Maricic, Svante Pääbo

**Affiliations:** ^a^Department of Evolutionary Genetics, Max Planck Institute for Evolutionary Anthropology, Leipzig D-04103, Germany; ^b^Human Evolutionary Genomics Unit, Okinawa Institute of Science and Technology, Okinawa 904-0495, Japan

**Keywords:** genetics, evolution, anthropology

## Abstract

The aryl hydrocarbon receptor (AHR) is a transcription factor that has many functions in mammals. Its best known function is that it binds aromatic hydrocarbons and induces the expression of cytochrome P450 genes, which encode enzymes that metabolize aromatic hydrocarbons and other substrates. All present-day humans carry an amino acid substitution at position 381 in the AHR that occurred after the divergence of modern humans from Neandertals and Denisovans. Previous studies that have expressed the ancestral and modern versions of AHR from expression vectors have yielded conflicting results with regard to their activities. Here, we use genome editing to modify the endogenous *AHR* gene so that it encodes to the ancestral, Neandertal-like AHR protein in human cells. In the absence of exogenous ligands, the expression of AHR target genes is higher in cells expressing the ancestral AHR than in cells expressing the modern AHR, and similar to the expression in chimpanzee cells. Furthermore, the modern human AHR needs higher doses of three ligands than the ancestral AHR to induce the expression of target genes. Thus, the ability of AHR to induce the expression of many of its target genes is reduced in modern humans.

## Significance

All present-day humans carry an amino acid substitution in the aryl hydrocarbon receptor (AHR), a transcription factor that induces the expression of cytochrome P450 genes. When the *AHR* gene is modified to encode the ancestral form of the protein seen in Neandertals and other non-modern human primates, the expression of AHR target genes is increased both in the absence of exogenous ligands and when ligands are added to the cells. Thus, compared to the ancestral variant present in archaic hominins and primates, the AHR in modern humans has a reduced ability to induce the expression of its target genes. This may represent an adaptation to exposure to polycyclic aromatic hydrocarbons in modern humans.

Genetic features that are present in all or almost all present-day humans, yet occur in the ancestral, chimpanzee-like state in our closest evolutionary relatives, the archaic hominins Neandertals and Denisovans, are of interest as some of them may be involved in phenotypic features that are unique to modern humans relative to archaic humans. According to one estimate (1), there are at least 31,389 such single nucleotide variants of which 96 change the amino acid sequence of 87 proteins. One of these proteins is the aryl hydrocarbon receptor (AHR). It is rare in that the ancestral variant of the AHR has to date not been found among hundreds of thousands of genomes sequenced (2).

AHR is a transcription factor that is involved in many physiological functions. Among its ligands are compounds produced endogenously in cells such as degradation products of tryptophan while other ligands stem from the microbiome and the environment (3). It also has a function in regulating stem cell maintenance, differentiation of cells, and immunity (4). Upon ligand binding in the cytosol, AHR enters the nucleus, dimerizes with an AHR nuclear translocator, and induces the transcription of target genes (5, 6). The main targets are cytochrome P450 genes that encode enzymes that metabolize aromatic hydrocarbons, that may originate from environmental exposures or be produced endogenously in the body, some of which may affect immune functions (e.g., ref. 7).

At position 381, the AHR protein of present-day humans carries a valine residue whereas the Neandertals, Denisovans, as well as other primates and vertebrates carry an alanine residue (8). Position 381 falls within the ligand binding pocket (9) and several studies have attributed differences between mouse and human AHR ligand binding to the valine-alanine difference at position 381 (10–12). However, recent studies where the archaic and modern versions of AHR were expressed from transfected plasmids have yielded contradicting results. Hubbard et al. (8) showed that the modern human version of AHR has reduced capability to induce the expression of the target genes *Cyp1a1* and *Cyp1b1* encoding cytochrome P450 family 1 subfamily A member 1 and subfamily B member 1, respectively, in rat cells when activated by three exogenous ligands. In contrast, Aarts et al. (13) treated human cells where either of the two AHR versions were overexpressed with the exogenous ligand 2,3,7,8-Tetrachlorodibenzo-p-dioxin and observed no differences between the two receptor forms with respect to the induction of the target gene *CYP1A1*. They suggested that the previous results might be an artifact stemming from the expression of human AHR in rat cells.

To overcome the potential problems of species incompatibilities as well as the expression of unphysiologically high amounts of AHR from expression vectors, we use genome editing to revert the valine at position 381 back to the ancestral alanine in human cells. We find that one endogenous AHR ligand (kynurenic acid, KYNA), one ligand produced by the intestinal flora (indirubin), and one exogenous ligand (benzo(a)pyrene, B(a)P) have drastically reduced abilities to induce the expression of the target genes *Cyp1a1* and *Cyp1b1*. Furthermore, in the absence of exogenous ligands, the expression of 14 AHR target genes is higher in cells expressing the ancestral than the modern AHR and similar to chimpanzee cells for three target genes tested.

## Results

### Genome Editing.

We transfected the human embryonic stem cell line H9 with ribonucleoprotein complexes of *Acidaminococcus* sp. BV3L6 Cas12a protein and guide RNA as well as a single-stranded DNA donor carrying the ancestral substitution (hg19: 7:17375392 T>C) and a silent substitution (hg19: 7:17375405 A>C) to prevent repeated cutting by Cas12a (14). To increase editing efficiency, four compounds that modulate DNA repair pathways were added to the cell culture for 24 h after transfection (*SI Appendix*, *Methods*).

We isolated 299 clonal cell lines and amplified and sequenced their target sites in the *AHR* gene. 71 of the clones carried the substitution (hg19: 7:17375392 T>C) that will result in valine to alanine substitution at position 381 (AHR^A381^) in the AHR protein as well as the silent substitution in a homozygous form. Fourteen clones carried no substitutions and thus encode valine residues at position 381 (AHR^V381^), i.e. they had not been changed in the editing process. Of the remaining clones, one carried only the silent substitution on both alleles, eight carried the silent substitution on both alleles, but the ancestral variant only on one, and 130 clones carried small insertions or deletions or unintended point mutations on one or both alleles in addition to, or instead of, the intended substitution. The other clones failed to grow or showed mixed genotypes.

### Quality Controls.

To detect loss of heterozygosity due to gene conversion induced by the strand breaks that can occur when CRISPR/Cas is used to edit genomic sites (15), we sequenced the closest heterozygous positions up- and down-stream of the edited sites (*SI Appendix*, Fig. S1) in each of the 71 clones that carried only the intended substitutions (edited clone) and the 14 clones that carried no substitutions (unedited clone). None of the unedited clones showed any artifacts, whereas of 71 edited clones two clones had lost the heterozygous state either up- or down-stream of the edited sites and one had lost the heterozygous states both up- and down-stream.

To detect deletions located at the Cas12a site but large enough to delete one or both of the primer sites used to amplify and sequence the target site (15–17), we used droplet digital PCR to estimate the copy number of the target site (*SI Appendix*, Fig. S1). One unedited clone and ten edited clones carried a single copy of the target site suggesting that a deletion had occurred while the remaining clones carried two copies of the target site.

In total, 58 edited clones carried the “ancestral” alleles in a homozygous form and 13 unedited clones carried the “modern” alleles in a homozygous form while not being affected by loss of heterozygosity. To mitigate the effects of clone-to-clone variability that can be a problem when cell lines derived from single cells are analyzed (18), we pooled the edited “ancestralized” clones and the unedited clones that had gone through the editing procedure but not experienced any nucleotide changes. To exclude chromosomal aberrations that can accumulate in stem cells in cell culture (19, 20), we performed shallow DNA sequencing of the two cell populations and their “mother” cell line. No aneuploidies or deletions or duplications were detected (*SI Appendix*, Fig. S2).

**Fig. 1.**
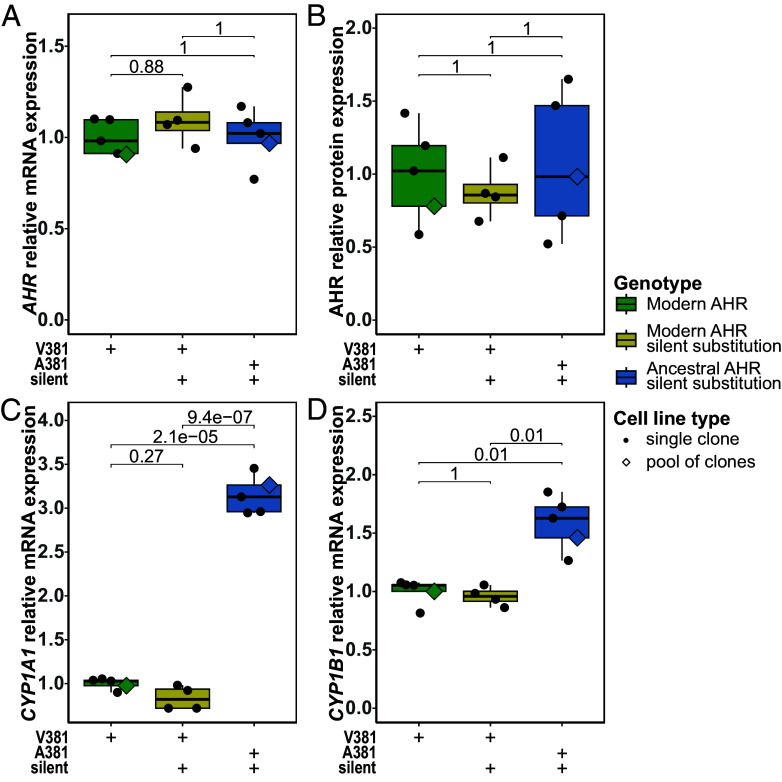
Relative *AHR, CYP1A1,* and *CYP1B1* expression levels in ancestral AHR^A381^ and modern AHR^V381^ cells. (*A*) *AHR* mRNA levels measured with RT-qPCR and normalized to *GUSB* mRNA levels. (*B*) AHR protein levels measured by western blot analysis and normalized to total protein amount per lane (pictures of membranes in *SI Appendix*, Fig. S3). (*C* and *D*) *CYP1A1* and *CYP1B1* mRNA levels measured with RT-qPCR and normalized to *GUSB* mRNA levels. Each point represents an independent clone and each diamond a pool of 13 AHR^V381^ clones or 58 AHR^A381^ clones that also carry the silent substitution. For mRNA levels, the mean of three qPCR replicates is shown. Boxes extend from the first to third quartile, the median is shown by the horizontal line and the whiskers extend from the box to the smallest and largest value. *P*-values determined by unpaired t-tests and Bonferroni correction for multiple testing.

To detect any effects on *AHR* mRNA or protein expression due to the ancestral or silent substitutions, we edited cells using donor molecules carrying only one or the other of these substitutions as well as both. The donor carrying only the ancestral substitution did not generate any correctly edited cells presumably due to repeated cutting by Cas12a (14). Among cells edited with the other two donors, we isolated nine clones carrying the silent substitution homozygously, four clones carrying both substitutions homozygously, and 16 unedited clones. We analyzed *AHR* mRNA expression by real-time quantitative PCR (RT-qPCR) in the pool of unedited clones, the pool carrying both mutations and four single clones carrying either the silent mutation, both mutations, or no mutations each. We found no differences in *AHR* mRNA expression (*P* = 0.88 to 1.0) (Fig. 1*A*). We analyzed AHR protein expression by western blots and similarly found no differences among the genotypes (*P* = 1.0) (Fig. 1*B* and *SI Appendix*, Fig. S3). Thus, the nucleotide changes introduced in the *AHR* gene do not affect mRNA or protein expression of AHR.

### AHR Target Gene Expression.

To test the function of the ancestral AHR^A381^ and the modern AHR^V381^, we analyzed the mRNA expression of *CYP1A1* and *CYP1B1* with RT-qPCR in the cells expressing the two AHR versions. Whereas cells carrying only the silent mutation express similar levels of *CYP1A1* and *CYP1B1* as not modified AHR^V381^-expressing cells (Fig. 1 *C* and *D*), AHR^A381^-expressing cells express approximately 3.2-fold more *CYP1A1* mRNA and 1.6-fold more *CYP1B1* mRNA than AHR^V381^-expressing cells. Thus, the ancestral AHR induces expression of its target genes more strongly in the absence of any externally added ligand.

As the silent substitution does not affect the expression of AHR or its target genes (Fig. 1 *A* and *B*), we performed subsequent experiments using the pools of clones expressing “modern” AHR^V381^ and clones carrying the two substitutions expressing the ancestral AHR^A381^, respectively, to mitigate the effect of clone-to-clone variability.

**Fig. 2.**
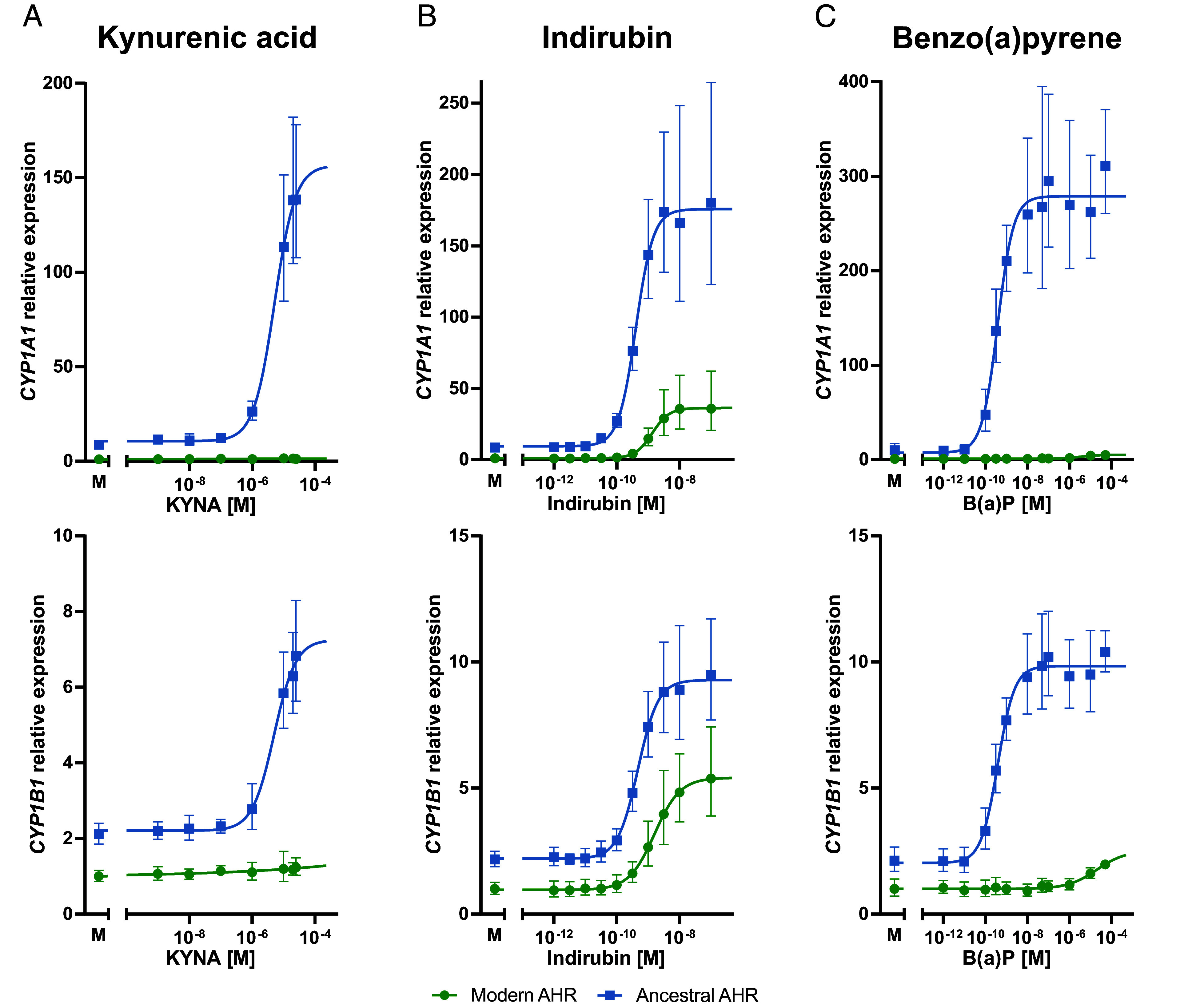
Expression of *CYP1A1* and *CYP1B1* after AHR ligand exposure in cells expressing AHR^A381^ and AHR^V381^. Ancestral and modern AHR*-*expressing cells were incubated with (*A*) KYNA, (*B*) indirubin, or (*C*) B(a)P for 4 h followed by quantification of *CYP1A1*, *CYP1B1,* and *GUSB*. Each panel shows relative *CYP1A1* and *CYP1B1* mRNA levels normalized to *GUSB* and relative to the mean expression in mock-treated (M, 0.05% DMSO) AHR^V381^ cells. Error bars show SDs of four independent ligand treatments. qPCR were performed in triplicates.

We incubated the cells with different concentrations of AHR ligands and measured *CYP1A1* and *CYP1B1* mRNA levels after 4 h. Kynurenic acid (KYNA), a degradation product of tryptophan, induces *CYP1A1* and *CYP1B1* expression at concentrations of approximately one micromolar in cells expressing the ancestral AHR^A381^. In stark contrast, in cells expressing the modern AHR^V381^, no clear induction of either target gene occurs (Fig. 2*A*) even at concentrations that are about 25-fold higher and exceed physiological concentrations in humans (21). Thus, whereas the ancestral AHR^A381^ is induced by kynurenic acid in human stem cells, the modern AHR^V381^ is not.

Indirubin, a compound produced by bacteria in the gut, induces *CYP1A1* and *CYP1B1* expression at similar concentrations in cells expressing both versions of AHR. However, the maximum expression of *CYP1A1* upon exposure to indirubin in cells expressing the ancestral AHR^A381^ is approximately fivefold higher than in cells expressing the modern AHR^V381^ (Fig. 2*B*) while the maximum expression of *CYP1B1* is approximately 1.7-fold higher. The half maximal effective concentration (EC_50_) of indirubin for AHR^A381^ is 400 pM (CI: 340 to 460 pM) and 470 pM (CI: 410 to 530 pM) for *CYP1A1* and *CYP1B1*, respectively. For AHR^V381^, the EC_50_ is 1,330 pM (CI: 1,250 to 1,420 pM) and 1,600 pM (CI: 1,500 to 1,700 pM) for *CYP1A1* and *CYP1B1*, respectively.

Benzo(a)pyrene (B(a)P), a polycyclic aromatic hydrocarbon generated by incomplete combustion of organic substances, induces *CYP1A1* and *CYP1B1* at concentrations that are at least five orders of magnitude lower in cells expressing the ancestral AHR^A381^ than in cells expressing the modern AHR^V381^ (Fig. 2*C*). In fact, for the modern AHR^V381^, only at B(a)P concentrations as high as 1 micromolar, *CYP1A1* and *CYP1B1* mRNA levels are slightly induced. Thus, the ancestral AHR^A381^ is more sensitive to benzo(a)pyrene than the modern AHR^V381^.

In conclusion, the modern AHR^V381^ induces the two target genes tested less strongly in the absence of any added exogenous ligand. The modern AHR^V381^ is much less sensitive to benzo(a)pyrene and to kynurenic acid and at high concentrations of indirubin, the modern AHR^V381^ induces much lower expression of target genes than AHR^A381^ (Fig. 2).

### Transcriptome-Wide Effects of AHR^A381^.

**Fig. 3.**
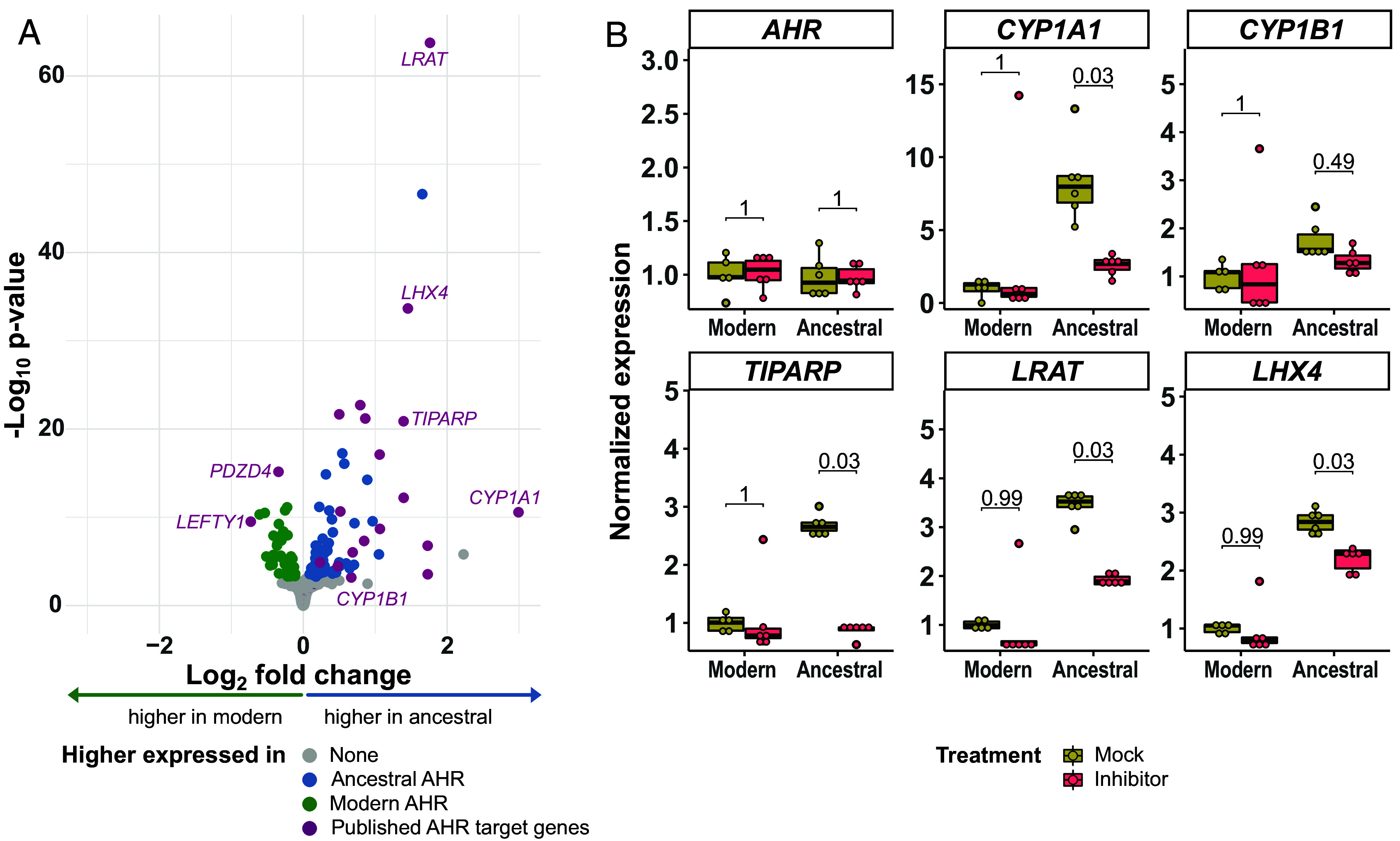
Expression of AHR target genes in cells with AHR^A381^ and AHR^V381^. (*A*) RNA sequencing results of ancestral AHR^A381^ versus modern AHR^V381^ cells, not treated with AHR ligands. Log_2_ expression difference between cells with ancestral and modern AHR is plotted versus −log_10_ raw *P*-value (Wald-test) of five independent cultures of cells with a dot for each detected gene. Genes with a Benjamini–Hochberg-corrected *P*-value <0.05 are highlighted in green and blue, as higher expressed in AHR^V381^ or AHR^A381^ cells, respectively. Known AHR target genes in H9 cells (22) are highlighted in purple. The names of genes mentioned in the text are indicated. (*B*) Relative expression of *AHR* and AHR target genes *CYP1A1*, *CYP1B1*, *TIPARP*, *LRAT,* and *LHX4* in RNA sequencing data of AHR^A381^ and AHR^V381^ cells mock-treated (0.05% DMSO) or treated with the AHR inhibitor CH-223191 (5 µM) for 4 h. Five cultures of treated and mock-treated cells were analyzed (dots) and expression values normalized to mean of mock-treated AHR^V381^ cells. Each box extends from the first to third quartile, the median is shown by the horizontal line and the whiskers extend from the box to the smallest and largest value which is not further than 1.5* interquartile range from the first or third quartile. *P*-values determined by Wilcoxon-rank tests and Bonferroni correction for multiple testing.

In order to gauge the effects of the alanine to valine substitution at position 381 in AHR on the transcriptome at large, we prepared RNA and cDNA libraries from six batches of the cell pools expressing AHR^A381^ and AHR^V381^, respectively, and sequenced 15 to 22 million molecules per library. At a false discovery-adjusted *P*-value of <0.05, 117 genes differ in expression between the two cell lines (Fig. 3*A*). Of these, 71 are higher expressed in the cells expressing the ancestral AHR^A381^ and 46 are higher expressed in the cells expressing the modern AHR^V381^.

Among the 117 genes, 16 have been previously described to be affected by exposure to 10 nM 2,3,7,8-Tetrachlorodibenzo-p-dioxin (TCDD), another AHR ligand, in H9 cells (22). Of these, two genes (*LEFTY1, PDZD4*) are less expressed upon TCDD exposure and both are lower expressed in the cells expressing the ancestral AHR^A381^ than in those expressing the modern AHR^V381^. The other 14 genes are higher expressed upon TCDD exposure and they are all higher expressed in the ancestralized AHR^A381^ cell line than in the modern AHR^V381^ cell line. Thus, the modern AHR^V381^-expressing cells express target genes less strongly than the ancestral AHR^A381^-expressing cells.

To investigate whether the expression of target genes in the H9 cells is indeed dependent on AHR activity, we treated the AHR^A381^ and AHR^V381^ cells with 5 µM CH-223191, an AHR inhibitor, for 4 h, and then performed RNA sequencing. The expression of *CYP1A1* as well as three additional AHR target genes (*TIPARP, LRAT, LHX4*) are all reduced in AHR^A381^-expressing cells (*P* < 0.05) (Fig. 3*B*). In the AHR^V381^-expressing cells, target gene expression is very low also in the absence of the inhibitor but tends to be further reduced upon treatment with the inhibitor, albeit not significantly so. Although the level of reduction of mRNA expression upon CH-223191 treatment depends on the half-life of the mRNA, which is likely to differ among the transcripts analyzed, the results show that the expression of these genes depends on AHR at least to a large extent. Overall, of the 117 genes whose expression is changed in ancestral AHR^A381^- compared to modern AHR^V381^-expressing cells without any ligand treatment, 20 genes change their expression upon treatment with the inhibitor in the ancestral AHR^A381^-expressing cells (*SI Appendix*, Fig. S4*B*) and do so in the same direction, whereas none of those genes change their expression in modern AHR^V381^-expressing cells (*SI Appendix*, Fig. S4*A*). Thus, at least a portion of the genes that differ in expression between cells expressing the ancestral AHR^A381^ and the modern AHR^V381^ are genes whose expression is dependent on AHR.

To test the transcriptome-wide response to exposure to an AHR ligand, we treated AHR^A381^- and AHR^V381^-expressing cells with 10 nM indirubin and analyzed their transcriptomes after 4 h. In AHR^A381^ cells, a total of 28 and three genes are up- and down-regulated, respectively, relative to mock treated cells. In AHR^V381^ cells, 34 and one gene are up- and down-regulated, respectively (*SI Appendix*, Fig. S5 *A* and *B*). Twenty-one genes change their expression in both cell lines (*SI Appendix*, Fig. S5*C*). Without ligand treatment, one of these genes is lower expressed and 20 genes are higher expressed in AHR^A381^ cells compared to AHR^V381^ cells. Upon indirubin treatment, the former gene reduces its expression and the other 20 genes increase their expression in both cell lines. For 16 of the 21 genes, the changes in expression upon indirubin treatment are stronger in AHR^V381^ cells than in AHR^A381^ cells. However, for 20 out of the 21 genes, the absolute expression levels are higher in AHR^A381^ cells after indirubin exposure. Thus, whereas the response of AHR^V381^ cells to indirubin is stronger in relative terms, AHR^A381^ induces genes to a greater absolute extent.

We also treated the AHR^A381^- and AHR^V381^-expressing cells with 50 nM B(a)P, the other ligand for which the modern AHR showed a response for *CYP1A1* and *CYP1B1* albeit a very small one (Fig. 2). In this case, 25 and four genes are up- and down-regulated in AHR^A381^ cells whereas no genes are significantly up- and down-regulated in AHR^V381^ cells (*SI Appendix*, Fig. S5 *D* and *E*).

In conclusion, the greater transcriptional response observed for *CYP1A1* and *CYP1B1* in AHR^A381^- than in AHR^V381^-expressing cells is representative of the transcriptional response of other genes in cells expressing the ancestral and modern versions of AHR.

### AHR Target Gene Expression in Humans and Chimpanzees.

We next used two previously published datasets on gene expression in human and chimpanzee stem cells to ask whether expression of AHR target genes in chimpanzee cells is more similar to the cells carrying the ancestralized AHR^A381^ or to the unmodified human cells.

**Fig. 4.**
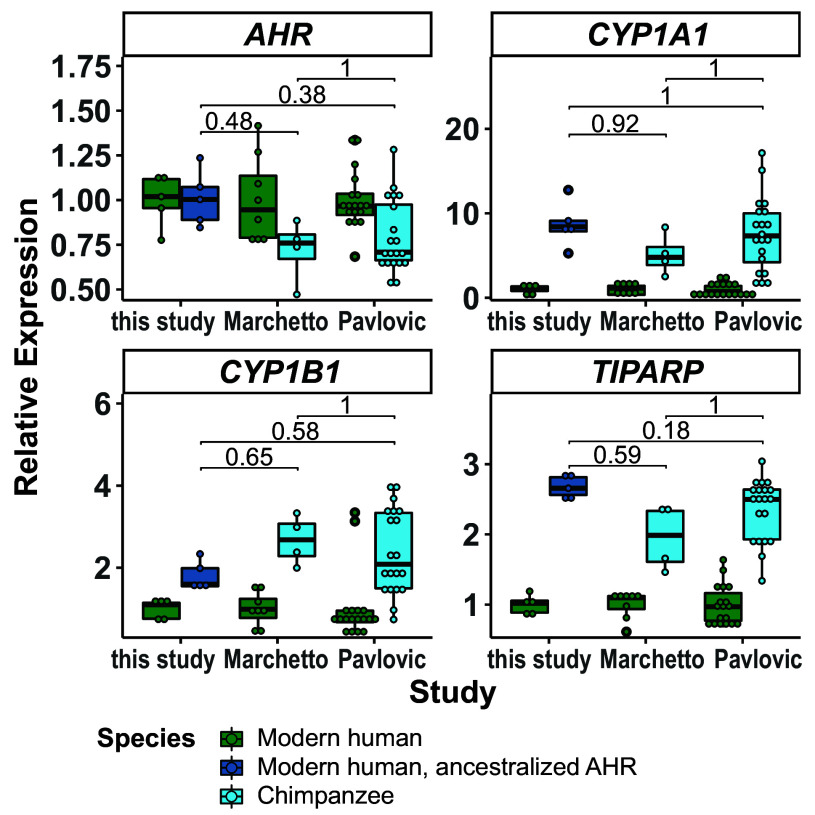
Expression of *AHR* mRNA and its target genes in human and chimpanzee cells and human cells expressing ancestralized AHR. Relative expression of *AHR* and the AHR target genes *CYP1A1*, *CYP1B1,* and *TIPARP* in AHR^A381^ and AHR^V381^ cells of this study and in two published datasets [Marchetto et al. (23) and Pavlovic et al. (24)] both containing RNA sequencing data of human and chimpanzee stem cells. Expression values are normalized to the expression of the respective genes in human (AHR^V381^) cells per study. Individual cultures are shown by dots. Each box extends from the first to the third quartile, the median is shown by the horizontal line and the whiskers extend from the box to the smallest and largest value which is not further than 1.5* interquartile range from the first or third quartile. *P*-values comparing the gene expression in human or chimp AHR^A381^ expressing cells between studies determined with the Wilcoxon-rank test and Bonferroni correction for multiple testing.

One study by Marchetto et al. (23) uses two chimpanzee iPSC lines, three human iPSC lines, and two human ESC lines, whereas the other study by Pavlovic et al. (24) uses 10 chimpanzee iPSC and nine human iPSC lines. *AHR* mRNA expression tends to be lower in the chimpanzee cells than in the human cells (*P* = 0.22 and *P* = 0.015 in the two studies, respectively). In the chimpanzee cells, the expression of the three AHR target genes *CYP1A1*, *CYP1B1,* and *TIPARP* are higher than in the human. Furthermore, in several cases, the relative expression differences between human and chimpanzee cells for these genes are similar in magnitude to the expression differences between unmodified human cells and human cells that carry the ancestral AHR^A381^ (Fig. 4).

Thus, the differences in target gene expression observed between present-day humans and chimpanzees are compatible with that the alanine to valine substitution at position 381 would be responsible for much of the differences in expression of these AHR target genes seen between present-day humans and chimpanzees.

## Discussion

Genomic changes that occurred and became fixed or nearly fixed in modern humans since their divergence from the lineage leading to Neandertals and Denisovans about half a million years ago are of interest as some of them will underlie phenotypes unique to modern humans. However, the fact that these changes are fixed among present-day humans makes it impossible to study their phenotypic consequences by association with phenotypes in the present-day population, leaving model systems as the only practical approach. A case in point is the alanine to valine substitution at position 381 in the AHR, which has so far not been seen in any present-day human. This change of one hydrophobic amino acid to another one in the ligand binding domain may be expected to have a rather mild effect (e.g., AlphaMissense pathogenicity score: 0.0585, classification: benign). However, it may be expected that many evolutionary amino acid substitutions would have subtle effects. Because AHR is involved in many physiological functions, many of which may be unknown, we decided to study this substitution with respect to the ability of AHR to activate its target genes.

Previous studies of AHR variants have expressed the protein from transfected plasmids in cells where expression may be very high (13) and may overwhelm components needed for biological effects, or in cells that do not express AHR (8) and that may thus lack components needed for its effects. In contrast, we used genome editing to introduce the substitution in the endogenous *AHR* genes in human H9 cells that express AHR where it is involved in maintaining the stem cell-like state of the cells (25). Also in contrast to previous studies, we changed only the amino acid at position 381, as this is the only coding variant in *AHR* that is fixed among modern humans. We also introduced a silent nucleotide substitution to prevent recutting and showed that these modifications do not affect *AHR* mRNA or protein expression (Fig. 1).

Several observations show that the alanine to valine substitution at position 381 decreases the ability of AHR to affect the expression of its target genes, at least in human stem cells. First, when no ligands are added to the cell culture, we find that the target genes *CYP1A1* and *CYP1B1* are higher expressed in cells carrying the ancestralized form of AHR than in the unmodified cells (Fig. 1). This is also the case for several other genes whose expression is affected by AHR (Fig. 3*A*). Strikingly, among those genes, 14 genes, which are known to be up-regulated by dioxin exposure (22), are higher expressed in the cells carrying the ancestral version of AHR whereas the two genes that are down-regulated by dioxin treatment are lower expressed. Second, upon exposure of the cells to kynurenic acid and benzo(a)pyrene the ancestral form of AHR induces *CYP1A1* and *CYP1B1* expression efficiently while the modern human AHR does not do so, or does so only at vastly higher concentrations (Fig. 2 *A* and *C*). However, the modern version of AHR is not devoid of any function because when exposed to indirubin, target genes are induced albeit at a lower level than is the case for the ancestral AHR (Fig. 2*B*). Furthermore, out of 20 genes that increase their expression upon indirubin exposure, 19 genes do so to a higher level in cells expressing the ancestral AHR and the single gene that reduces its expression in both cell lines does so to a greater extent (*SI Appendix*, Fig. S5 *A*–*C*). This is compatible with a recent cryoelectronmicroscopic study of the structure of human AHR, which suggests that the valine residue at position 381 is contacting indirubin (26). Upon exposure to benzo(a)pyrene, 25 and four genes are up- and down-regulated in cells expressing the ancestral AHR, respectively, and no genes change their expression significantly in the cells expressing the modern protein (*SI Appendix*, Fig. S5 *D* and *E*). Thus, modern human AHR affects the expression of many or all of its target genes to a lesser extent than the ancestral version in human stem cells. It should be noted, though, that the response may depend on factors that are specific to certain cell types and that the response may therefore vary among cell types. For example, although no induction of *CYP1A1* by kynurenic acid is detectable in the stem cells (Fig. 2), it is able to induce *CYP1A1* expression in some human cells (27).

Interestingly, the AHR target genes *CYP1A1, CYP1B1,* and *TIPARP* are also higher expressed in chimpanzee stem cell lines than in human stem cell lines (Fig. 4). This suggests that the alanine to valine substitution at position 381 is largely responsible for the difference in AHR target gene expression seen between human and chimpanzee stem cells.

It is striking that the alanine to valine substitution in AHR is absent among all archaic individuals studied to date yet present in all present-day humans sequenced to date, including, for example, almost 1.2 million genomes and exomes in the gnomAD database (28, 29). This is in contrast to most modern human-specific genetic variants, for which ancestral alleles exist in low frequencies in present-day populations due to archaic gene flow or persistence of ancestral variants (see https://bioinf.eva.mpg.de/catalogbrowser) (2). It has been speculated that the reduction of the AHR pathway activity was an adaptation toward a lifestyle dependent on the use of fire (8), as smoke from the burning of wood is a rich source of polycyclic aromatic hydrocarbons (30, 31). Support for this comes from the observation that fish exposed to high levels of AHR ligands from industrial pollution accumulate mutations in genes of the AHR signaling pathway leading to reduced expression of AHR target genes (32, 33), for example by a reduced ability of AHR to bind ligands. However, the apparently complete absence of the ancestral form of AHR in present-day people raises the possibility that the valine substitution at position 381 in the AHR may have some additional hitherto unknown function. Further studies of the function of the ancestral and modern forms of AHR in other cell types such as liver (34), lung (35), the central nervous system (36), and the immune system (37) may thus be warranted.

## Supplementary Material

Appendix 01 (PDF)

## Data Availability

RNA sequencing data are deposited in the GEO database under accession number GSE210514 (38).
