# Supplementary material for: The modern human aryl hydrocarbon receptor is more active when ancestralized by genome editing
Source: Proc Natl Acad Sci U S A. 2024 May 13;121(22):e2402159121. doi: 10.1073/pnas.2402159121 (PMC11145187; doi:10.1073/pnas.2402159121)
Supplement: Supplementary file 1 — Appendix 01 (PDF) [file pnas.2402159121.sapp.pdf]

The modern human aryl hydrocarbon receptor is more active when ancestralized by genome editing

Nelly Helmbrecht[1]*(ORCID ID: 0000-0002-5384-8430), Martin Lackner[1] (ORCID ID: 0000-0001-8450-7295), Tomislav Maricic[1] (ORCID ID: 0000-0003-3267-0474), Svante Pääbo[1,2]* (ORCID ID: 0000-0002-4670-6311)

1. Department of Evolutionary Genetics, Max Planck Institute for Evolutionary Anthropology, D-04103 Leipzig, Germany

2. Human Evolutionary Genomics Unit, Okinawa Institute of Science and Technology, Okinawa 904-0495, Japan.

* *Correspondence to nelly_helmbrecht@eva.mpg.de or paabo@eva.mpg.de*

Supplementary Methods

Cell culture

The human embryonic stem cell line H9 (female, WiCell Research Institute, ethics permit AZ 3.04.02/0118) was maintained at 37°C and 5% CO_2 in a humidified incubator with daily media changes. Cells were grown on Matrigel matrix (Corning, 35248) coated dishes in mTeSR1 medium (StemCell Technologies, 85850) supplemented with MycoZap Plus-CL (Lonza, VZA-2012) and were passaged when 80% confluency was reached. For passaging, cells were washed with DPBS followed by incubation with TrypLE Express (1x, Thermo Fisher Scientific, 12605010) or Accutase solution (Sigma-Aldrich, A6964) for 5-8 min at 37°C. The reaction was stopped by adding twice the volume of medium, followed by centrifugation (3min, 300g) to remove the enzymes. The cell pellet was resuspended in medium and cells plated at 1:5 to 1:20 dilution. To reduce apoptosis, the medium was supplemented with 10 µM of Rho-associated protein kinase inhibitor Y-27632 (Sigma-Aldrich, 688000) for 24 h after passaging.

Human immortalized myelogenous leukemia cells (K562) cells were cultured in IMDM media (Thermo Scientific, 12440053) supplemented with 10 % FBS (Sigma-Aldrich, F2442), HyClone MEM Non-essential Amino Acids Solution (Cytiva, SH30238.01) and MycoZap Plus-CL (Lonza, VZA-2012) at 37°C and 5% CO2 in a humidified incubator. Media was changed every second day and cells were passaged once per week.

Cell cultures were tested for mycoplasma contamination on a regular basis. DNA was extracted from cells using the Gentra Puregene Cell Kit (Qiagen, 158767) and used for PCR based mycoplasma detection with the Venor GeM Classic kit (Minerva Biolabs, 11-1250).

Generation of genome edited cell lines

gRNA and ssODN Donor design. Cas12a was used to generate edited cell lines carrying ancestral variant (hg19: 7:17375392 T>C, V381A). A single-stranded DNA donor (ssODN) was designed to contain the desired substitution, an additional silent mutation (hg19: 7:17375405 A>C) to prevent re-cutting by Cas12a or both. The silent mutation does not change the resulting amino acid, using a codon with similar codon usage frequency as the original (1). Homology arms of the ssODN were 45 nt on both sides of the desired substitution for Cas12a editing. Guide RNAs and ssODN were purchased from Integrated DNA Technologies (IDT, Coralville, USA), for sequences see supplementary table S1.

Genome editing. Transfection was performed according to the gRNA manufacturer's instructions except for the following alterations: Ribonucleoproteins were formed by incubating 252 pmol recombinant Acidaminococcus sp. BV3L6 Cas12a (Cpf1) Ultra protein (IDT, 10001272) together with 320 pmol guide RNA for 20 min at room temperature. Approximately 1 million cells in 100 µl Nucleofector solution for

human stem cells (Lonza, VVPH-5022) were mixed with the ribonucleoproteins, with 78 pmol Alt-R Cpf1(Cas12a) electroporation enhancer (IDT, 1076300), and 200 pmol ssODN and transfected using the Nucleofector 2b Device (Lonza) and program B-16.

For the editing experiment from which clones were pooled, the medium was supplemented with 10 µM Y-27632 and 2 µM M3814 (MedChemExpress, HY-101570), 0.01 µM Trichostatin A (SIGMA, T8552), 0.5 µM MLN4924 (Adooq BioScience, A11260), and 5 µM NSC 15520 (ChemBridge, 6048069) for 24 h after nucleofection (2, 3). For the editing experiments from which only individual clones were generated, the nucleofection solution contained 160 pmol of POLQ siRNA (Horizon Discovery, ON-TARGET plus Human POLQ siRNA - SMART 10721) and 320 pmol of POLQ siRNA 765 (Integrated DNA Technologies, DsiRNA hs.Ri.POLQ.13.8). After nucleofection, the medium was supplemented with 10 µM Y-27632 and 2 µM M3814 for 24 h (4).

Clone generation. Clonal colonies that were later pooled were generated from single cells using the CellRaft AIR System (Cell Microsystems) and clonal colonies from the other set of editing experiments were generated using the Single Cell Printer (Cytena). For the CellRaft Air system, cells were grown in StemFlex (Thermo Fisher Scientific, A3349401) supplemented with CloneR (Stemcell Technologies, 05889) for 48 h prior to cell sorting. Clonal lines were generated with the CellRaft AIR System (Cell Microsystems) according to the manufacturer’s instructions. Briefly, a single cell solution was generated with Accutase and 4000-8000 cells in StemFlex supplemented with CloneR were seeded on each reservoir of a 200 micron Quad Reservoir CytoSort Array (Cell Microsystems) coated with 0.5 % iMatrix 511 (Matrixome, 892021). Cells were counted with the Countess II FL automated cell counter (Invitrogen). The medium was changed to mTeSR1 on day four. Arrays were scanned at least every other day to identify rafts with growing colonies that originate from a single cell. These rafts were transferred to Matrigel matrix coated 96 well culture plates with conditioned mTeSR1 on day six by the CellRaft AIR System. The medium was changed every second day until day ten and daily afterwards.

For the Single Cell Printer, cells were grown in StemFlex supplemented with CloneR for 24 h prior to generating a single cell suspension with Accutase. After centrifugation, cells were resuspended in warm DPBS. 50 µL cell suspension was transferred to a super hydrophobic cartridge (Cytena, CZ.XS.CAR.012) and single cells sorted in 96 well culture plates coated with 0.5 % iMatrix 511 containing 60 µL conditioned StemFlex supplemented with CloneR. On day three after sorting, 60 µL per well StemFlex supplemented with CloneR was added to the cells, on day six the media was changed to StemFlex. From day 8, mTeSR1 medium was changed daily.

Illumina library preparation and sequencing. Cells were dissociated with TrypLE Express or Accutase, pelleted and resuspended in 20 µl QuickExtract (Epicentre, QE0905T). Cell suspensions were mixed and

incubated for 10 min at 65 °C, 5 min at 68 °C and 5 min at 98 °C to generate single-stranded DNA. To amplify genomic regions of interest, 3 µl of this suspension were used per 25 µl PCR reaction with 500 nM primer and KAPA2G Robust HotStart PCR Kit with buffer B (Kapa Biosystems, KK5024) and the PCR ran in a C1000 Thermal Cycler (Bio-Rad) with the following cycling conditions: 95 °C 3 min, 35x (95 °C 15 s, 60 °C 15 s, 72 °C 15 s), 72 °C 1 min. Primers were tailed with adapters for Illumina sequencing and obtained from IDT (supplementary table S1). In a subsequent PCR reaction, P7 and P5 flow cell adapters and sample-specific indices were added (5). 0.5 µl product of the first PCR were used per 25 µl reaction with Phusion High-Fidelity PCR Master Mix with HF Buffer (Thermo Scientific, F-531L), 400 nM primer and the following cycling conditions: 98 °C 30 s, 25x (98 °C 10 s, 58 °C 20 s, 72 °C 20 s), 72 °C 5 min. Successful amplification was verified by size separating agarose gel electrophoresis using 2 % EX Agarose Gels (Thermo Fisher Scientific, G4010–11). PCR amplicons were pooled and purified using Solid Phase Reversible Immobilization (SPRI) beads (6). Multiplexed libraries were sequenced 2x 150 bp (+ 2x 7 bp index) on a MiSeq System (Illumina). Base calling was done using Illumina's Bustard and adapters were trimmed using leeHom (7).

Three regions per clone were amplified and sequenced: the region targeted in genome editing to determine the editing outcome and the closest heterozygous positions upstream and downstream of that site to exclude loss of heterozygosity or deletions bigger than that range (see section "Detection of heterozygous indels").

Amplicon sequence analysis. CRISPResso2 (8) was used for initial quantification of genome editing outcomes. Analysis was restricted to a 20 bp window from each gRNA cleavage site and only paired reads were considered that shared at least 70 % sequence similarity with the wild type sequence. Substitutions other than the expected ones were ignored to filter out amplification and sequencing errors.

SAMtools (9) was used to verify the genotypes of clones indicated by CRISPResso2. If the clear majority of reads of a given clone consisted of one sequence or two sequences in equal ratio, the genotype of this clone was determined by aligning these sequences to their respective reference sequences (BLAST global align (10)).

Detection of heterozygous indels. A combination of different methods was applied to detect loss of heterozygosity and unintended heterozygous insertions or deletions that cannot be detected through amplification and sequencing of the region targeted during editing alone (Suppl. Fig. 1).

The closest heterozygous positions upstream and downstream of the editing target site were amplified and sequenced. Clonal cell lines with only one allele at either or both positions were discarded, as this indicates a loss of heterozygosity or deletion extending to the sequenced position.

To cover duplications or deletions smaller than the two sequenced positions but too big to be detected by sequencing of the editing target site, droplet digital PCR (ddPCR) with primer and probe binding sites within the sequenced target site region was used to identify copy number variation of the target site. One µl heat activated QuickExtract cell suspension and 200 nM of each primer and fluorescent probe for target and reference sites (IDT, sequences: Suppl. Table S1) were mixed with ddPCR Supermix for Probes (no dUTP) (Bio-Rad, 1863024). The 20 µl reaction together with droplet generation oil for probes (Bio-Rad, 1863005) was applied to DG8 Cartridges (Bio-Rad, 1864007) for partitioning the reaction mix in a QX200 Droplet Generator (Bio-Rad, 1864002) according to the manufacturer's instructions. The PCR run with the following cycling conditions: 95 °C 5 min, 40x (95 °C 30 sec, 60 °C 1 min), 98 °C 5 min. The fluorescent droplets were quantified with the QX200 Droplet Reader (Bio-Rad, 1864003) and the copy number of the target site in comparison to the reference was calculated using QuantaSoft software (version 1.7.4, Bio-Rad Laboratories).

Pooling of clones. Fifty-eight precisely edited clones carrying both substitutions as well as 13 control clones that passed through the same process but remained wild type were generated from one editing experiment. To reduce the effects of clone-to-clone variability, all clones with the same genotype were pooled. For clone pooling, clones were grown to approximately 70% confluency in 96 well plates. Single-cell suspensions were generated with Accutase, mixed and approximately 100,000-200,000 cells seeded on 6 well plates. Pools were cultured for a maximum of seven passages.

Shallow DNA sequencing of cell lines. Shallow DNA sequencing was performed for pooled cell lines. DNA was extracted from cell pellets of edited, unedited, and mother cell lines using the DNeasy Blood & Tissue Kit (Qiagen), and Illumina libraries were constructed as described in (6), but with double indexing (5). Multiplexed libraries were sequenced and base calling was performed as for amplicons. Demultiplexed libraries were mapped to GRCh38 with default settings, duplicates were marked (GATK MarkDuplicatesSpark) and reads mapping to problematic regions (11) were removed. For each 10MB window of the genome, the number of uniquely mapped reads was counted using Mosdepth (12). The output .gz files were loaded into a Python script that generated Suppl. Fig. 2.

Immunoblotting. Cultured H9 and K562 cells were lysed with RIPA buffer (Thermo Scientific, 89901) supplemented with Halt Protease-Inhibitor-Cocktail (Thermo Scientific, 78429) followed by sonication. The protein concentration was determined with a BCA-Protein Assay (Sigma-Aldrich, BCA1-1KT). Eight µg protein were electrophoresed on a 4-12 % NuPAGE Bis-Tris Gel (Invitrogen, NP0322) using MOPS Running Buffer (Invitrogen, NP0001) and transferred to a PVDF membrane (Invitrogen, LC2002). The total protein was fluorescently labeled using No-Stain Protein Labeling Reagent (Invitrogen, A44449), imaged with the iBright FL1500 Imaging System (Thermo Scientific) and quantified with the iBright Analysis

Software (Thermo Scientific). After blocking with EveryBlot Blocking Buffer (BioRad, 12010020), the membranes were incubated with 1:1000 dilutions of anti-AHR-D5S6H (Cell Signaling Technology, 83200) antibody overnight at 4°C. Subsequently, the membranes were incubated with a 1:10,000 dilution of HRP conjugated Goat-anti-rabbit-IgG (Invitrogen, 31460) secondary antibody. Chemiluminescence from the SuperSignal West Dura Extended Duration Substrate (Thermo Scientific, 37071) was imaged with the iBright FL1500 Imaging System. Chemiluminescence band intensities were quantified with the iBright Analysis Software and normalized to the total lane protein amount.

AHR, CYP1A1 and CYP1B1 relative quantification and ligand assays

Benzo[a]pyrene (B1760), indirubin (PHL89716-10MG), kynurenic acid (K3375) and CH-223191 (C8124) were purchased from Sigma-Aldrich, dissolved in DMSO (Invitrogen, D12345) to generate stocks 2000x the final concentrations used in the ligand assays and stored at -20°C.

Four days prior to cell harvest, a single cell solution was prepared with Accutase and the cells were counted with the Countess II FL automated cell counter. 3000 cells per well were seeded in 96 well plates and cultured under normal cell culture conditions for four days, with the last media change 24h before cell harvest. Ligands were added to a final concentration of 0.05% DMSO in the culture medium for four hours similar to other work (13-15). Cell harvest, reverse transcription and quantitative real-time PCR was performed with the TaqMan Fast Advanced Cells-to-CT Kit (Thermo Fisher Scientific, A35374) following the manufacturer's instructions. Cells were lysed directly in the cell culture dish by adding 50 µl Lysis Solution with 1% DNase I. Lysis suspension was mixed and incubated for 5 min at room temperature before mixing with 5 µl Stop Solution. 20 µl of this crude cell lysate was used in a 50 µl reverse transcription reaction with 25 µl 2X Fast Advanced RT Buffer, 2.5 µl 20x Fast Advanced RT Enzyme Mix, and 5 µl RNase free water. Reverse transcription was performed in a C1000 thermal cycler for 30 min at 37 °C followed by enzyme inactivation for 5 min at 95°C. Relative mRNA abundance of *CYP1A1*, *CYP1B1*, and for several samples also *AHR* was measured in a 20 µl multiplexed quantitative PCR reaction with 10 µl TaqMan Fast Advanced Master Mix, 7 µl cDNA, and 1 µl CYP1A1, CYP1B1 or AHR and GUSB primer-probe-mix each (250nM probe and 900nM primer final concentration, obtained from IDT (CYP1A1, GUSB) or Thermo Fisher Scientific (CYP1B1, AHR), sequences: Suppl, Table S1). Quantitative PCR was run in triplicates on a CFX96 Cycler (Bio-Rad) with the following cycling conditions: 55 °C 5 min, 95 °C 5 min, 45x (95 °C 5 s, 60 °C 15 s, 72 °C 15 s, plate read). Cell cultures: n=4. For primer and probe sequences, see Suppl. Table S1.

Cq values were determined with Bio-Rad Laboratories' CFX-Manager (version 3.1) and relative expression values obtained by normalizing to beta-glucoronidase (GUSB) Cq values. Target gene

expression values were estimated by comparison to the mean of relative expression values of the respective gene in untreated wild type cells. The data were plotted and analyzed using GraphPad Prism (version 8.4.3). Nonlinear regression (curve fit) using the model “[Agonist] vs. response – Variable slope (four parameters)” and least squares regression were used performed to determine minimal (bottom) and maximal (top) response values, half maximal effective concentrations (EC50) and corresponding 95% confidence intervals.

RNA extracts for RNA sequencing

Cells were counted with the Countess II FL automated cell counter and 100,000 cells were seeded four days prior to cell harvest. Medium was changed daily, with the last media change 23 h before cell harvest and the ligands were added 4 h before cell harvest. Final ligand concentrations were 50 nM benzo(a)pyrene, 10 nM indirubin, and 5 µM CH-223191. Final concentration of DMSO was 0.05% for all samples. For each batch, cells from both genotypes and all treatments were harvested in parallel.

Cells were harvested and RNA extracted using the RNeasy Plus Mini Kit (Qiagen, 74134) following the manufacturer's instructions. Briefly, approximately one million cells were lysed with buffer RLT supplemented with 40 mM DL-Dithiothreitol (Sigma-Aldrich, 43816) and homogenized using QIAshredder columns (Qiagen, 74134). Genomic DNA was removed by column-binding. RNA integrity of total RNA was determined with capillary gel electrophoresis performed with the 2100 Bioanalyzer (Agilent) and the RNA Nano 6000 assay (Agilent, 5067-1511). All RNA samples generated in this study had a RNA integrity number of 10. N=6.

RNA sequencing libraries and data processing

RNA-Seq libraries were prepared with the Collibri Stranded RNA Library Prep Kit for Illumina Systems with Human/Mouse/Rat rRNA Depletion Kit (Thermo Fisher Scientific, A39003096) according to the manufacturer’s instructions. All libraries were prepared in parallel. Briefly, 300 ng total RNA were depleted from rRNA and the RNA was then fragmented, adapters hybridized and ligated to the RNA, followed by reverse transcription and cDNA amplification in an indexing PCR with 11 cycles. The library size distribution of the amplified cDNA was assessed on a 2100 Bioanalyzer using the High Sensitivity DNA Kit (Agilent, Cat. No. 5067-4626). The following libraries were excluded from the analysis due to insufficient library quality: AHR_WT_DMSO_6 and AHR_WT_Indirubin_3. Indexed libraries were pooled and sequenced 125 bp single-end (+ 8 bp index) with 15 - 22 million reads per library on an Illumina HiSeq 2500 (Illumina). Illumina’s Bustard was used for base calling and adapters were trimmed using

leeHom (7). Reads were then aligned to the human reference (GRCh37.87) using tophat2 (v2.0.14) using default parameters (16). Aligned reads were assigned to protein coding genes using the featureCounts software (17). Differential expression was calculated using read counts per gene as input for the multi-factor model implemented in DESeq2 (18). Factors included in this analysis were genotype (states: wild type and ancestral), batch, and ligand treatment. Genes with multiple-testing corrected (Benjamini-Hochberg) p-value below 0.05 were defined as differentially expressed. For volcano plots, log2 fold changes were shrunken using apeglm (19).

Analysis of human and chimpanzee iPSCs

RPKM values were downloaded for Marchetto, *et al.* (20) (GSE47626) and read counts for Pavlovic, *et al.* (21) (GSE110471) from NIH GEO database. RPKM values were calculated for Pavlovic, *et al.* (21) and untreated ancestral and modern cells from the current study using DESeq's fpkm function (18). RPKM values for *AHR*, *CYP1A1*, *CYP1B1* and *TIPARP* were each normalized to the mean (wild type) human RPKM value for the respective gene per study. Statistical analysis was performed using unpaired two-samples Wilcoxon test and Bonferroni correction for multiple testing.

Supplementary Tables and Figures

Supplementary Table S1: Sequences of guide RNA, ssODN, primers, fluorescent probes and Taqman-assays. For ssODN, ancestral variant and silent mutation are highlighted in bold. Primers for sequencing are tailed with adapters for Illumina sequencing (first 33 bases for forward primers, first 34 bases for reverse primers). All oligonucleotides acquired from IDT, except for TaqMan-assays, which were obtained from Thermo Fisher Scientific.

Name	Sequence
Cas12a guide RNA, variable part	TGCTTACGTTAGTGGTCTCTG
ssODN (ancestral mutation (hg19: 7:17375392 T>C, V381A) and silent mutation (hg19: 7:17375405 A>C))	TGCACGCCTGCTTTATAAAAATGGAAGACCAGATTATATCATTG**C**AACTCAGAGACC**C**CTAACGTAAGCACAAATAATGTTTCCTGTTTT
ssODN (ancestral mutation (hg19: 7:17375392 T>C, V381A))	TGCACGCCTGCTTTATAAAAATGGAAGACCAGATTATATCATTG**C**AACTCAGAGACC**A**CTAACGTAAGCACAAATAATGTTTCCTGTTTT
ssODN (silent mutation (hg19: 7:17375405 A>C))	TGCACGCCTGCTTTATAAAAATGGAAGACCAGATTATATCATTG**T**AACTCAGAGACC**C**CTAACGTAAGCACAAATAATGTTTCCTGTTTT
AHR_target_for	ACACTCTTTCCCTACACGACGCTCTTCCGATCTACTGGAGAAAGTGGCATGAT
AHR_target_rev	GTGACTGGAGTTCAGACGTGTGCTCTTCCGATCTAGTCTGCTTGAATTATTTTTATGCT
AHR_upSNP_NGS_for	ACACTCTTTCCCTACACGACGCTCTTCCGATCTATGTCTCAGGCCTTCACATT
AHR_upSNP_NGS_rev	GTGACTGGAGTTCAGACGTGTGCTCTTCCGATCTACAGCATGCTATCGTACTGA
AHR_downSNP_NGS_for	ACACTCTTTCCCTACACGACGCTCTTCCGATCTGTGAACTCATTTGCTGGAGG
AHR_downSNP_NGS_rev	GTGACTGGAGTTCAGACGTGTGCTCTTCCGATCTTCTTGGACATACGTCAGGAT
AHR_ddPCR_for	AGTTTTCCGGCTTCTTACAA
AHR_ddPCR_rev	AGGAAACATTATTTGTGCTTACGT
AHR_ddPCR_probe	FAM-CACGCCTGC-ZEN-TTTATAAAAATGGAAGAC-IABkFQ
CNVreference_ddPCR_for	GCAACAGCAATTGGCAGC
CNVreference_ddPCR_rev	CAGCGATTGGACAGGAAGTG
CNVreference_ddPCR_probe	HEX-AGCAGCAGCAGCATCTGCTCAGCCT-BHQ_1
CYP1A1_qPCR_for	TGGAGATTGGGAAAAGCATGA
CYP1A1_qPCR_rev	GAACCTTCCCTGATCCTTGTG
CYP1A1_qPCR_probe	HEX-TCTTGGAGG-ZEN-TGGCTGAGGTACTGA-IABkFQ
CYP1B1_TaqMan_assay	FAM-Hs00164383_m1
AHR_TaqMan_assay	FAM-Hs00169233_m1
GUSB_qPCR_for	GTTTTTGATCCAGACCCAGATG

GUSB_qPCR_rev	GCCCATTATTCAGAGCGAGTA
GUSB_qPCR_probe	Cy5-TGCAGGGTTTCACCAGGATCCAC-IAbRQSp

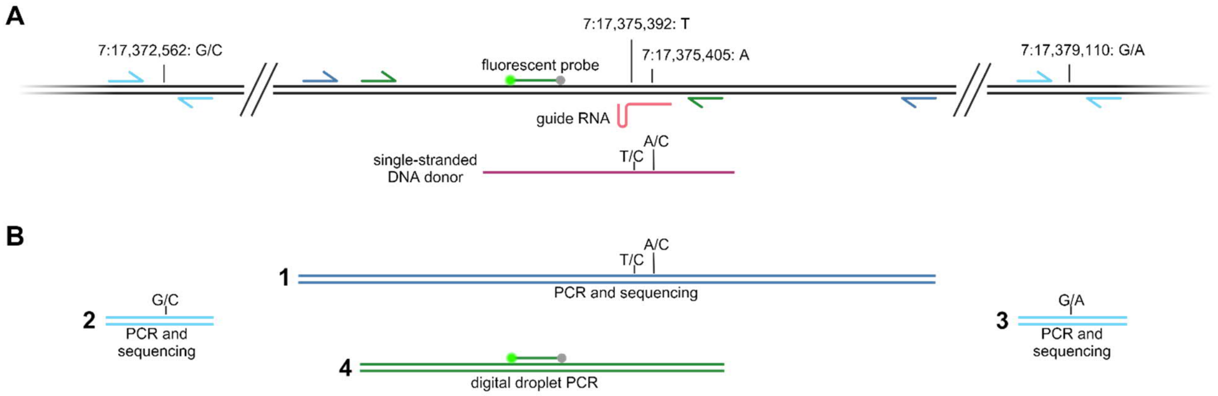

Supplementary Fig. 1: Schematic illustration of approaches used to detect unintended on-target effects by CRISPR/Cas12a genome editing. (A) The genomic *AHR* region binding sites of primers used for amplification (arrows), fluorescent probe, guide RNA and the single-stranded DNA donors indicated, as well as the two sites edited in different experiments. (B) 1: A region 220 bp–region around the edited site was sequenced to detect the ancestral mutation (hg19: 7:17,375,392 T>C, V381A), silent mutation (hg19: 7:17,375,405 A>C) and any unintended changes. 2, 3: Two heterozygous position ~3 kb upstream (7:17,372,562) and ~4 kb downstream (7:17,379,110) of the edited site were amplified and sequenced to detect loss of heterozygosity and larger deletions. 4: Digital droplet PCR was used to detect deletions or duplications that would include the target site but not extend to the other heterozygous positions.

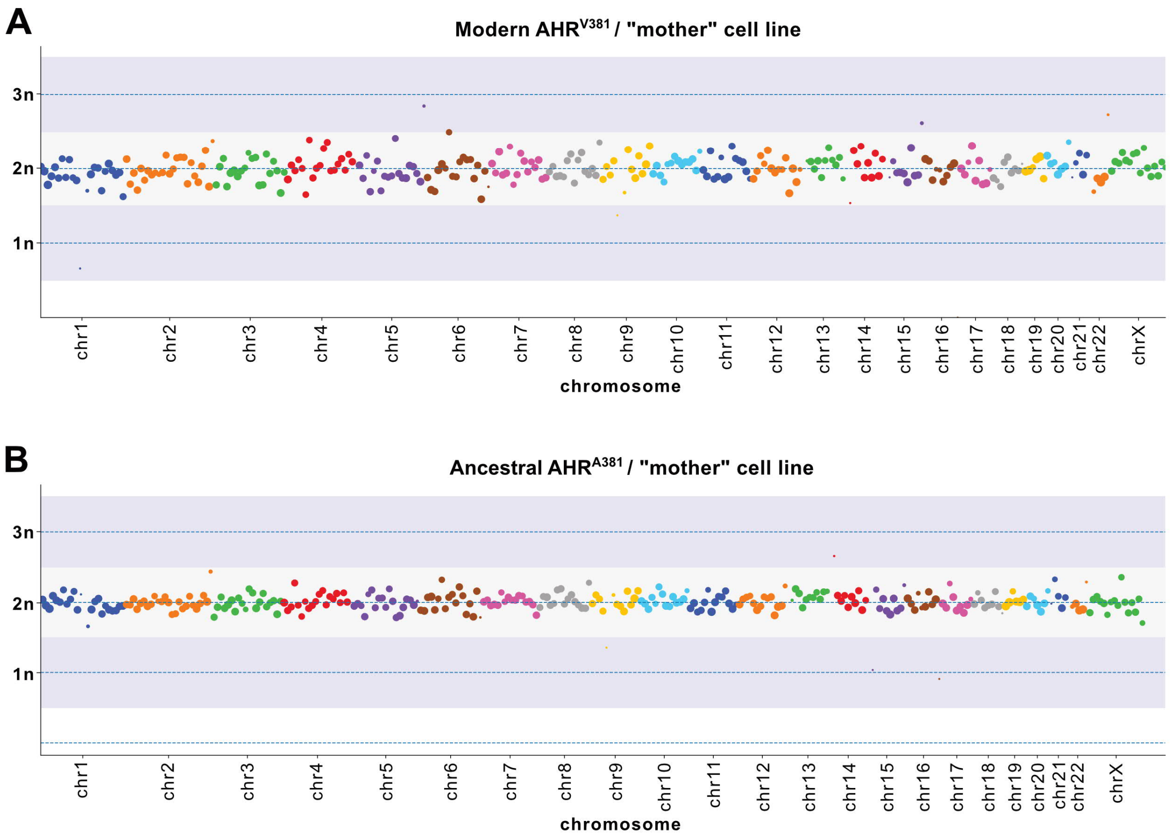

Supplementary Fig. 2: Ratio of reads mapping to (A) modern AHR^{A381} and (B) ancestral AHR^{V381} cells and their "mother" cell line along chromosomes. Circles represent 10MB windows and the size of each circle is proportional to the number of reads in the window. On the Y-axis, 2n is calculated as the ratio of all the reads in the edited or unedited pool and "mother" cell line, whereas 3n is 1.5*2n and 1n is 0.5*2n. It is noteworthy that only a few of the smaller circles, which correspond to a lower number of sequences and are therefore more vulnerable to stochasticity, are noticeably distant from the 2n line.

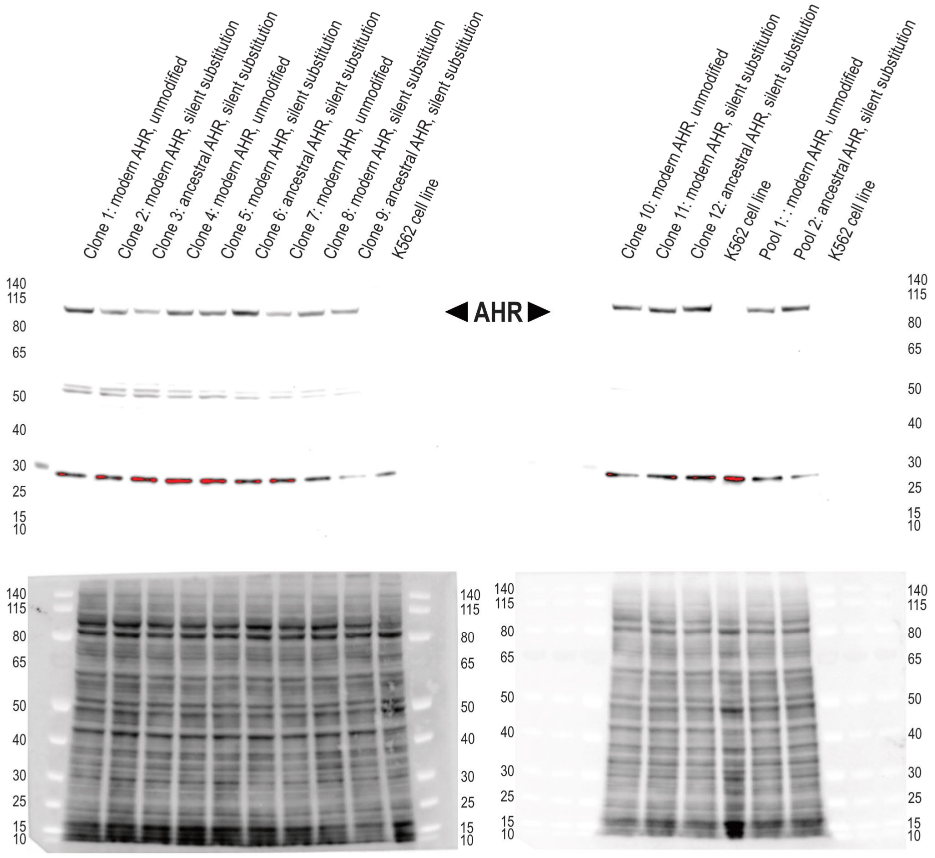

Supplementary Fig. 3: AHR protein levels in ancestral AHR^{A381} and "modern" AHR^{V381} cells. Relative AHR protein levels measured by western blot analysis and corresponding total protein amount on lane. Per genotype, four cellular clones are analyzed, as well as an ancestral AHR^{A381} pool of 58 clones and a "modern" AHR^{V381} pool of 13 clones. K562 cells do not express AHR and serve to identify unspecific bands. Ladder: PageRuler Prestained Protein Ladder, 10-180 kDa (Thermo Scientific, 26616).

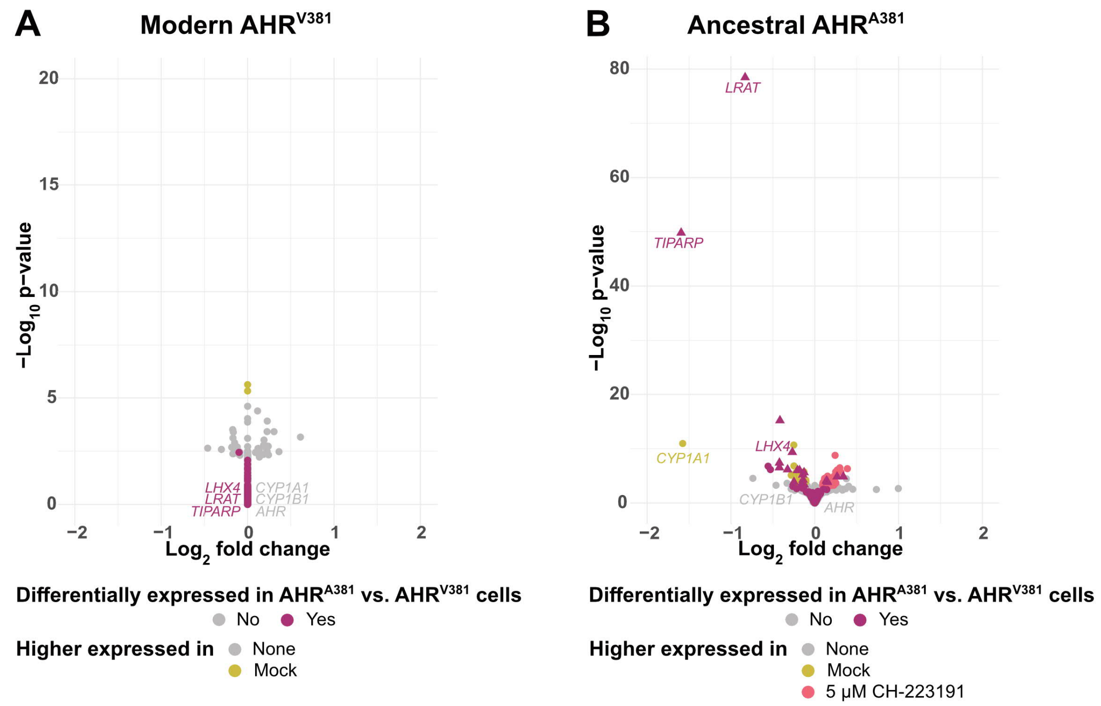

Supplementary Fig. 4: Transcriptome-wide effects in AHR^{A381} and AHR^{V381} cells treated with the AHR inhibitor CH-223191. (A) and (B) RNA sequencing results of (A) AHR^{A381}- or (B) AHR^{V381}-expressing cells treated with 5 µM CH-223191 for four hours versus mock (0.05% DMSO) treated cells. For each detected gene, a dot is plotted with the $\log_2$ expression difference between CH-223191 and mock treated cells and $-\log_{10}$ of the associated raw p-value (Wald-test). Red dots indicate significantly upregulated and yellow dots downregulated genes upon inhibitor treatment (Benjamini-Hochberg adjusted p-value <0.05). Purple dots indicate genes differentially expressed between AHR^{A381} and AHR^{V381} cells without ligand treatment (Fig. 3A) (Benjamini-Hochberg adjusted p-value <0.05), triangles indicate genes that are furthermore differentially expressed between CH-223191 and mock treated cells. N = 5.

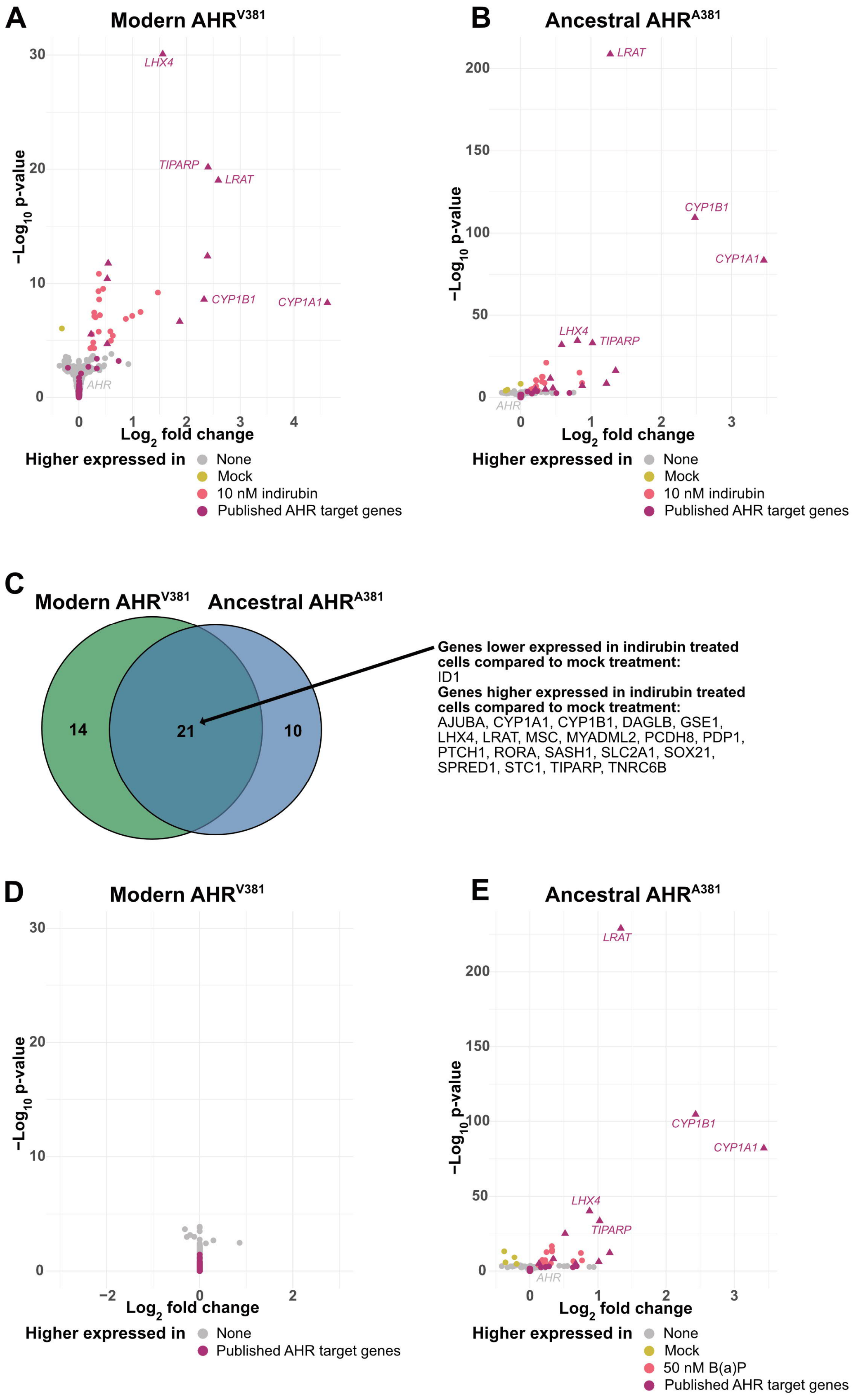
A
Modern AHR^{V381}
−Log$_{10}$ p-value
Log$_2$ fold change
LHX4
TIPARP
LRAT
CYP1B1
CYP1A1
AHR
Higher expressed in
None
Mock
10 nM indirubin
Published AHR target genes
B
Ancestral AHR^{A381}
−Log$_{10}$ p-value
Log$_2$ fold change
LRAT
CYP1B1
CYP1A1
LHX4
TIPARP
AHR
Higher expressed in
None
Mock
10 nM indirubin
Published AHR target genes
C
Modern AHR^{V381}
Ancestral AHR^{A381}
14
21
10
Genes lower expressed in indirubin treated cells compared to mock treatment:
ID1
Genes higher expressed in indirubin treated cells compared to mock treatment:
AJUBA, CYP1A1, CYP1B1, DAGLB, GSE1, LHX4, LRAT, MSC, MYADML2, PCDH8, PDP1, PTCH1, RORA, SASH1, SLC2A1, SOX21, SPRED1, STC1, TIPARP, TNRC6B
D
Modern AHR^{V381}
−Log$_{10}$ p-value
Log$_2$ fold change
Higher expressed in
None
Published AHR target genes
E
Ancestral AHR^{A381}
−Log$_{10}$ p-value
Log$_2$ fold change
LRAT
CYP1B1
CYP1A1
LHX4
TIPARP
AHR
Higher expressed in
None
Mock
50 nM B(a)P
Published AHR target genes

Supplementary Fig. 5: Transcriptome-wide effects in cells expressing AHR^{A381} and AHR^{V381} treated with indirubin or B(a)P. (A) and (B) RNA sequencing results of (A) AHR^{A381} and (B) AHR^{V381} cells treated with 10 nM indirubin for four hours versus mock (0.05% DMSO) treated cells. For each detected gene, a dot is plotted with the $\log_2$ expression difference between indirubin and mock treated cells and –$\log_{10}$ of the associated raw p-value (Wald-test). Red dots indicate significantly upregulated and yellow dots downregulated genes upon ligand treatment (Benjamini-Hochberg adjusted p-value <0.05). Known AHR target genes (22) are highlighted in purple, triangles indicate known AHR target genes that are differentially expressed in ligand-treated cells. The names of genes mentioned elsewhere in the text are indicated. N = 5. (C) Number and overlap of genes differentially expressed (p-value <0.05, Benjamini-Hochberg adjusted Wald test) upon 10 nM indirubin treatment in AHR^{V381} and AHR^{A381} cells. (D) and (E) RNA sequencing results of (D) AHR^{A381} and (E) AHR^{V381} cells treated with 50 nM B(a)P for four hours versus mock (0.05% DMSO) treated cells. Plotting details as for (A) and (B). N=5.

References

1. J. Athey *et al.*, A new and updated resource for codon usage tables. *BMC Bioinform.* **18**, 391 (2017).
2. S. Riesenberg, T. Maricic, Targeting repair pathways with small molecules increases precise genome editing in pluripotent stem cells. *Nat. Commun.* **9**, 2164 (2018).
3. S. Riesenberg *et al.*, Simultaneous precise editing of multiple genes in human cells. *Nucleic Acids Res.* **47**, e116 (2019).
4. S. Riesenberg *et al.*, Efficient high-precision homology-directed repair-dependent genome editing by HDRobust. *Nat. Methods* **20**, 1388-1399 (2023).
5. M. Kircher, S. Sawyer, M. Meyer, Double indexing overcomes inaccuracies in multiplex sequencing on the Illumina platform. *Nucleic Acids Res.* **40**, e3 (2012).
6. M. Meyer, M. Kircher, Illumina sequencing library preparation for highly multiplexed target capture and sequencing. *Cold Spring Harb. Protoc.* **2010**, pdb.prot5448 (2010).
7. G. Renaud, U. Stenzel, J. Kelso, leeHom: adaptor trimming and merging for Illumina sequencing reads. *Nucleic Acids Res.* **42**, e141 (2014).
8. K. Clement *et al.*, CRISPResso2 provides accurate and rapid genome editing sequence analysis. *Nat. Biotechnol.* **37**, 224–226 (2019).
9. H. Li *et al.*, The sequence alignment/map format and SAMtools. *Bioinformatics* **25**, 2078-2079 (2009).
10. S. F. Altschul, W. Gish, W. Miller, E. W. Myers, D. J. Lipman, Basic local alignment search tool. *J. Mol. Biol.* **215**, 403–410 (1990).
11. H. M. Amemiya, A. Kundaje, A. P. Boyle, The ENCODE Blacklist: Identification of Problematic Regions of the Genome. *Sci. Rep.* **9**, 9354 (2019).
12. B. S. Pedersen, A. R. Quinlan, Mosdepth: quick coverage calculation for genomes and exomes. *Bioinformatics* **34**, 867-868 (2018).
13. T. D. Hubbard *et al.*, Divergent Ah receptor ligand selectivity during hominin evolution. *Mol. Biol. Evol.* **33**, 2648-2658 (2016).
14. B. C. Spink, M. M. Hussain, B. H. Katz, L. Eisele, D. C. Spink, Transient induction of cytochromes P450 1A1 and 1B1 in MCF-7 human breast cancer cells by indirubin. *Biochem. Pharmacol.* **66**, 2313-2321 (2003).
15. C. Genies *et al.*, The extreme variety of genotoxic response to benzo[a]pyrene in three different human cell lines from three different organs. *PLoS One* **8**, e78356 (2013).
16. D. Kim *et al.*, TopHat2: accurate alignment of transcriptomes in the presence of insertions, deletions and gene fusions. *Genome Biol.* **14**, R36 (2013).
17. Y. Liao, G. K. Smyth, W. Shi, featureCounts: an efficient general purpose program for assigning sequence reads to genomic features. *Bioinformatics* **30**, 923-930 (2014).
18. M. I. Love, W. Huber, S. Anders, Moderated estimation of fold change and dispersion for RNA-seq data with DESeq2. *Genome Biol.* **15**, 550 (2014).
19. A. Zhu, J. G. Ibrahim, M. I. Love, Heavy-tailed prior distributions for sequence count data: removing the noise and preserving large differences. *Bioinformatics* **35**, 2084-2092 (2019).
20. M. C. N. Marchetto *et al.*, Differential L1 regulation in pluripotent stem cells of humans and apes. *Nature* **503**, 525-529 (2013).
21. B. J. Pavlovic, L. E. Blake, J. Roux, C. Chavarria, Y. Gilad, A comparative assessment of human and chimpanzee iPSC-derived cardiomyocytes with primary heart tissues. *Sci. Rep.* **8**, 15312 (2018).
22. I. Teino *et al.*, Impact of AHR ligand TCDD on human embryonic stem cells and early differentiation. *Int. J. Mol. Sci.* **21**, 9052 (2020).
